# Reliable Fault Diagnosis of Bearings Using an Optimized Stacked Variational Denoising Auto-Encoder

**DOI:** 10.3390/e24010036

**Published:** 2021-12-24

**Authors:** Xiaoan Yan, Yadong Xu, Daoming She, Wan Zhang

**Affiliations:** 1 School of Mechatronics Engineering, Nanjing Forestry University, Nanjing 210037, China; 2 School of Mechanical Engineering, Southeast University, Nanjing 211189, China; ydxu@seu.edu.cn; 3 School of Mechanical Engineering, Jiangsu University, Zhenjiang 212013, China; 1000005461@ujs.edu.cn; 4 Department of Automation, Nanjing University of Information Science and Technology, Nanjing 210044, China; zhangwan@nuist.edu.cn

**Keywords:** stacked variational denoising auto-encoder, seagull optimization algorithm, rolling bearing, fault diagnosis

## Abstract

Variational auto-encoders (VAE) have recently been successfully applied in the intelligent fault diagnosis of rolling bearings due to its self-learning ability and robustness. However, the hyper-parameters of VAEs depend, to a significant extent, on artificial settings, which is regarded as a common and key problem in existing deep learning models. Additionally, its anti-noise capability may face a decline when VAE is used to analyze bearing vibration data under loud environmental noise. Therefore, in order to improve the anti-noise performance of the VAE model and adaptively select its parameters, this paper proposes an optimized stacked variational denoising autoencoder (OSVDAE) for the reliable fault diagnosis of bearings. Within the proposed method, a robust network, named variational denoising auto-encoder (VDAE), is, first, designed by integrating VAE and a denoising auto-encoder (DAE). Subsequently, a stacked variational denoising auto-encoder (SVDAE) architecture is constructed to extract the robust and discriminative latent fault features via stacking VDAE networks layer on layer, wherein the important parameters of the SVDAE model are automatically determined by employing a novel meta-heuristic intelligent optimizer known as the seagull optimization algorithm (SOA). Finally, the extracted latent features are imported into a softmax classifier to obtain the results of fault recognition in rolling bearings. Experiments are conducted to validate the effectiveness of the proposed method. The results of analysis indicate that the proposed method not only can achieve a high identification accuracy for different bearing health conditions, but also outperforms some representative deep learning methods.

## 1. Introduction

Mechanical equipment is widely used in all walks of life in modern society, where rolling bearings are the most common component of mechanical equipment. Due to the continuous influence of alternating impact force and load, rolling bearings are easily subject to varying degrees of fault, in different positions. Once bearings are damaged, it will inevitably cause mechanical equipment to stop work, bringing about economic loss and even causing personnel casualties [1]. Therefore, accurate diagnosis of bearing faults is of great significance in ensuring the safe and reliable operation of mechanical equipment [2]. That is, it is very valuable to develop effective bearing fault diagnosis technology for the field of mechanical health monitoring.

With the rapid development of technologies such as sensors and industrial internet, the concept of intelligent diagnosis provides a new pathway for feature learning and the intelligent recognition of bearing faults [3,4]. Many intelligent diagnosis techniques have been successfully applied to bearing fault diagnosis and have achieved a good identification result. For instance, Gunerkar et al. [5] adopted wavelet transform (WT) to extract bearing fault features from time domain signals, whereafter the extracted features were input into a back-propagation neural network (BPNN) to identify multiple bearing faults. Jin et al. [6] used wavelet packet transform (WPT) and power spectrum analysis to obtain fault features, and then adopted a support vector machine (SVM) to classify the fault types. Li et al. [7] combined variational mode decomposition (VMD) and approximate entropy to extract bearing fault feature information, and an improved kernel extreme learning machine (KELM) was used to identify bearing fault types. However, the above-mentioned methods are all shallow learning methods, which have a high requirement for expert experience and knowledge and artificial feature extraction. In other words, these shallow learning methods have many disadvantages. Specifically, in the above methods, WT and WPT can effectively extract nonlinear time–frequency characteristics, but require the selection of suitable basis functions when processing complex vibration signals. BPNN has a nonlinear mapping and self-learning abilities, but easily falls into local minimization and has a slow convergence speed in the learning process [7]. SVM can efficiently solve the problem of high-dimensional nonlinear decision making, but it is difficult to select kernel parameters and penalty parameters and is significantly affected by faulty samples. VMD is an adaptive signal decomposition method and has good anti-noise performance. However, its mode number needs to be estimated according to prior knowledge. Beyond these issues, although KELM is fast in feature learning and fault diagnosis, its stability is relatively weak.

To avoid the above problems and forgo the dependence on artificial feature extraction, increasingly, many deep learning methods (e.g., deep belief network (DBN) [8], convolutional neural network (CNN) [9], auto-encoder (AE) [10] and stacked denoising auto-encoder (SDAE) [11]) are being presented to autonomously mine the representative diagnostic information hidden in the raw data and have received great attention in intelligent fault diagnosis. Shao et al. [12] presented a novel method, called an adaptive deep belief network (DBN), with a dual-tree complex wavelet packet (DTCWPT) to process bearing vibration signals and achieve fault diagnosis of rolling bearings. Liu et al. [13] adopted an ensemble convolutional neural network (ECNN) model, obtaining accurate and robust results in bearing fault diagnosis under complex conditions. Zhang et al. [14] proposed a new ensemble deep contractive auto-encoder (EDCAE) to process the data collected in noisy environments and obtain accurate, intelligent diagnoses. Although the above-mentioned standard deep learning model is able to effectively deal with industrial, big-data scenarios, their training accuracy will be reduced when are faced with little sample data. Therefore, a new generative network model, named variational auto-encoder (VAE), was proposed by Kingma and Welling in 2013 [15], which could automatically learn more representative and discriminative fault features from the little collected sample data by using variational inference and reparameterization techniques. Specifically, in the VAE method, one constraint is added to the encoding network so that the generated latent variables roughly follow the standard normal distribution, by which recognition accuracy could be greatly improved [16]. To date, some important achievements of VAE have been made in the field of fault diagnosis. The specific research progress of VAE is summarized in Table 1, including the research method, research problem and data source. It can be seen from statistics data provided in Table 1 that the existing VAE methods have good ability to solve different fault diagnosis problems, but they mainly focus on the problem of fault diagnosis at steady speeds and rely on human experience to select their network parameters without self-adaptation. Additionally, there are few studies investigating the robustness of VAE in the existing literature. In other words, due to fault features of the collected bearing vibration signal being easily submerged by environmental noises, it is very difficult to identify bearing faults by traditional older methods. Moreover, although the existing VAE methods have made some achievements in fault diagnosis, their feature-learning performances and anti-noise abilities may be reduced when existing VAE models are used to analyze bearing vibration data containing a lot of noise and nonlinear components. Therefore, the research focus of this paper is to find a new way to detect the health status of bearings. In other words, differently from the mentioned earlier methods, the method proposed in this paper mainly focuses to self-adaptive parameter selection and anti-noise performance improvements upon VAE models, at both steady and variable speeds.

In this study, to improve the anti-noise ability of VAE and adaptively select its network parameters, a new deep learning model, named optimized stacked variational denoising autoencoder (OSVDAE), is proposed for bearing fault diagnosis that can mine more representative fault information hidden in the raw bearing vibration data and establish accurately mapped relationships between it and the operating state of equipment. Specifically, the proposed OSVDAE method mainly consists of two stages (i.e., a parameter optimization stage and a feature learning stage). The major differences between our study and previous fault diagnosis studies can be summed up in two points. On the one hand, in the feature learning stage, considering a stacked denoising auto-encoder (SDAE) can not only improve the anti-noise ability of the auto-encoder, but also automatically learn more representative and discriminative fault feature information through stacking multiple denoising auto-encoder networks. Therefore, by integrating the advantages of SDAE and VAE, a stacked variational denoising autoencoder (SVDAE) is proposed for the feature learning of bearing faults. On the other hand, in the parameter optimization stage, a recently proposed intelligent optimizer, the seagull optimization algorithm (SOA) [17], which has a faster operating speed and convergence performance than some existing representative optimizers (e.g., particle swarm optimization (PSO), sine cosine algorithm (SCA), Genetic algorithm (GA) [18] and Grey wolf optimizer (GWO)), is adopted to automatically select the key parameters of the SVDAE model in this paper. The originality and novelties of this study can be summarized as follows:

(1) A novel deep learning model, named stacked variational denoising autoencoder (SVDAE), is proposed, which can effectively process bearing vibration data under noisy environment and obtain high-quality deep features.
(2) A new intelligent optimizer, named seagull optimization algorithm (SOA), is introduced to automatically determine the important parameters of SVDAE, which can avoid the empiricism and triviality of repeatedly manually adjusting parameters in deep learning methods.
(3) A data-driven fault diagnosis scheme based on an optimized stacked variational denoising autoencoder is proposed for bearing fault identification, which can improve the accuracy of bearing fault diagnosis.
(4) The effectiveness and superiority of the proposed method are demonstrated by experiments and comparisons between bearing fault diagnoses.

The rest of this paper is organized as follows. Section 2 introduces the variational autoencoder and reviews its application in fault diagnosis. Section 3 describes the contents related to the proposed method, including the variational denoising autoencoder, stacked variational denoising autoencoder and optimized stacked variational denoising autoencoder. Section 4 conducts two sets of experiments and comparisons to show the efficacy and superiority of the proposed method in bearing fault detection. Section 5 gives the conclusions of this paper.

## 2. Theoretical Background

In this section, the basic theory of VAE is firstly described, then we used a summary table to conduct the literature review on VAE in fault diagnosis.

### 2.1. Overview of Variational Auto-Encoder

**Figure 1:**
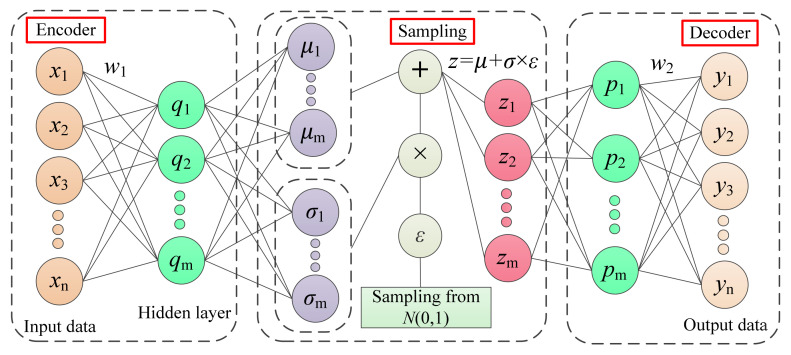
Architecture of the VAE model.

VAE is regarded as a new unsupervised learning algorithm with an encoder, sampling and a decoder that has a fast convergence rate and little overfitting. Figure 1 shows the architecture of VAE. As is apparent and differently from standard AE, the VAE model has an additional sampling layer beyond the encoder and decoder. In this way, in training the VAE model, the latent variable, *z,* with a Gaussian distribution can be obtained, the parameters of which (i.e., mean and variance) can be automatically learned by a network training process, such that new input data can be generated by the decoding for *z.* To make the generated data highly similar to the original data, here, maximized marginal distribution is conducted by

(1) $$p_{\theta }(x)=\int p_{\theta }(x/z)p_{\theta }(z)dz$$

where $p_{\theta }(\left.x\right|z)$ denotes the reconstruction of the original data, *x,* by the latent variable, *z*; $p_{\theta }(z)$ denotes the prior distribution of latent variable *z*.

Supposing that we have an approximation posterior, $q_{\varphi }(z\left|x\right.)$; the basic idea of the VAE model is to automatically learn a variational approximation posterior $q_{\varphi }(z\left|x\right.)$ to replace the real posterior distribution $p_{\theta }(\left.z\right|x)$, where φ and θ denote the parameters of the encoder and decoder, respectively. To achieve this, the loss function of VAE is calculated by the variational lower bound of likelihood function, which mainly consists of two items (see Equation (2)).

(2) $$L(\theta ,\varphi ;x^{\left(i\right)})=-D_{KL}(q_{\varphi }(z\left|x^{\left(i\right)}\right.)\left\|p_{\theta }(z)\right.)+E_{q_{\varphi }(z\left|x^{\left(i\right)}\right.)}[\log\;p_{\theta }(x^{\left(i\right)}\left|z\right.)]$$

where the first item $D_{KL}(q\left\|p)\right.$ represents the Kullback–Leibler divergence, the second item $E_{q}(\log\;p)$ means the reconstruction error. Supposing that $p_{\theta }(z)$ satisfies the standard normal distribution *N*(0, 1), $q_{\varphi }(\left.z\right|x)$ obeys the Gaussian distribution $N(\mu ,\sigma^{2})$, and the Kullback–Leibler divergence can be rewritten as

(3) $$-D_{\mathrm{KL}\;}[q_{\varphi }(\left.z\right|x^{\left(i\right)})\left\|p_{\theta }(z)\right.]=\frac{1}{2}\sum_{j=1}^{J}\left(1+\log\;{\left(\sigma_{j}^{\left(i\right)}\right)}^{2}-{\left(\mu_{j}^{\left(i\right)}\right)}^{2}-{\left(\sigma_{j}^{\left(i\right)}\right)}^{2}\right)$$

where *J* is the dimension of the latent variable *z,* $\mu_{j}^{\left(i\right)}$ and $\sigma_{j}^{\left(i\right)}$ represent the *j*-th element of $q_{\varphi }(\left.z\right|x)$. Meanwhile, the reconstruction error can be expressed as

(4) $$E_{q_{\varphi }(z\left|x^{\left(i\right)}\right.)}[\log\;p_{\theta }(x^{\left(i\right)}\left|z\right.)]=\frac{1}{L}\sum_{l=1}^{L}\log\;p_{\theta }(x^{\left(i\right)}\left|z^{\left(i,l\right)}\right.)$$

where *L* denotes the amount of sampling from $q_{\varphi }(\left.z\right|x)$. The reparameterization trick is adopted to update *z* under the rules of z=μ+ε⋅σ. Therefore, Equation (2) can be rewritten as

(5) $$L(\theta ,\varphi ;x^{\left(i\right)})=\frac{1}{2}\sum_{j=1}^{J}\left(1+\log\;{\left(\sigma_{j}^{\left(i\right)}\right)}^{2}-{\left(\mu_{j}^{\left(i\right)}\right)}^{2}-{\left(\sigma_{j}^{\left(i\right)}\right)}^{2}\right)+\frac{1}{L}\sum_{l=1}^{L}\log\;p_{\theta }(x^{\left(i\right)}\left|z^{\left(i,l\right)}\right.)$$

where $z^{\left(i,l\right)}=\mu^{\left(i\right)}+\sigma^{\left(i\right)}\varepsilon^{\left(l\right)}$ and $\varepsilon^{\left(l\right)}∼N(0,1)$. The goal of the VAE model is to minimize the loss function shown in Equation (2). Due to the addition of KL divergence into the loss function of the VAE model, the VAE model can not only effectively learn the underlying characteristics from the original data, but also can generate new sample data by sampling and decoding. Specific details of VAE can be found in the literature [15].

### 2.2. Literature Review on Variational Auto-Encoder in Fault Diagnosis

Since the VAE method’s appearance, many studies on VAE in fault diagnosis have been presented in the literature. Table 1 lists the current research circumstance of the VAE model in fault diagnosis. Specifically, Chen et al. [19] proposed a variational stacked autoencoder (VSAE) to adaptively extract fault features from angular domain signals and effectively diagnose valve train faults of a diesel engine under various operating conditions. Huang et al. [20] adopted a recurrent neural network (RNN)-based VAE method for realizing the dimensionality reduction of time series data and motor fault detection. Martin et al. [21] used a fully unsupervised deep VAE-based approach for dimensionality reduction and bearing fault diagnosis. Zhao et al. [22] combined a VAE and CNN model to handle small fault sample data and identify faults in rolling bearings. Wang et al. [23] applied a combination method, called conditional variational auto-encoder generative adversarial network (CVAE-GAN), to generate fault samples and diagnose imbalanced faults in a global gearbox. Yan et al. [24] proposed a variational autoencoder-based conditional Wasserstein GAN with gradient penalty (CWGAN-GP-VAE) to diagnose various faults for chillers. Tang et al. [25] combined the VAE model and deep variational information bottleneck (VIB) to monitor and identify quality-related and quality-unrelated faults. Costa et al. [26] proposed a semi-supervised recurrent variational autoencoder (RVAE) method to effectively address the diagnosis of atrial fibrillation (AF). Kim et al. [27] presented a fault diagnosis model to robustly process drift by modeling process drift with a variational autoencoder (VAE). Zhang et al. [28] presented a semi-supervised learning framework for bearing fault diagnosis using variational autoencoder (VAE)-based deep generative models. Chao et al. [29] used a knowledge-induced learning with adaptive sampling variational autoencoder (KIL-AdaVAE) for fault detection and the fault segmentation of complex safety-critical systems.

From to the literature survey, it can be seen that the studies mentioned above can solve the problems in fault diagnosis to a certain extent, but most of the above methods handle fault diagnosis problem at constant speed and select the parameters of the VAE model according to human experience. Additionally, the robustness of the above algorithm against noise is rarely studied. That is, the existing literature generally focuses on the application of VAE research to fault diagnosis under steady speeds, but little consideration is given to the concentrated surveying of parameter selection and the anti-noise capabilities of VAE models. Differently from these previous studies, this study mainly focuses to self-adaptive parameter selection and improving the anti-noise performance of a VAE model at constant speed and variable speed. Concretely, the novelty and contribution of this study can be summarized in three aspects:

(1) To improve the anti-noise capability of VAE model, a new deep learning model, named stacked variational denoising auto-encoder (SVDAE), is constructed by integrating stacked denoising techniques and a variational auto-encoder.
(2) To avoid the dependence of human experience of parameter selection of the existing VAE model, we adopted a novel optimizer, the seagull optimization algorithm (SOA), using its fast operation to adaptively select the parameters of SVDAE.
(3) To improve the accuracy of fault diagnosis, an intelligent diagnosis scheme based on optimized stacked variational denoising autoencoder (OSVDAE) is proposed that can effectively identify bearing faults under constant and variable speeds.

## 3. Proposed Methodologies

In this section, the basic theory of variational denoising auto-encoder (VDAE) is firstly presented, then a stacked variational denoising auto-encoder (SVDAE) is constructed on the basis of VDAE, and we use a novel meta-heuristic optimizer, the seagull optimization algorithm (SOA), to select the parameters of SVDAE. Finally, an intelligent bearing fault diagnosis scheme, based on optimized stacked variational denoising autoencoder (OSVDAE) is proposed.

### 3.1. Variational Denoising Auto-Encoder

**Figure 2:**
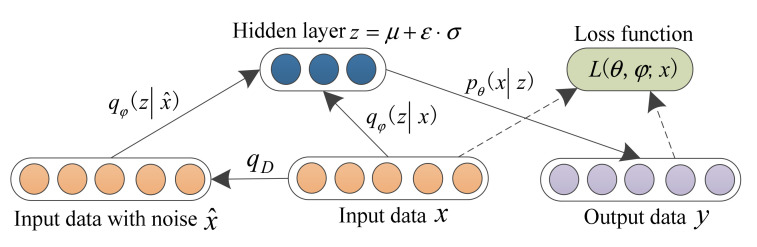
Architecture of the VDAE model.

Due to unstable working conditions and complex operating environments, when a local fault appears in rolling bearings, the resulting bearing vibration signal contains a mass of noise interference, which indicates that the obtained identification accuracy will be not too high if traditional AE is used to process the noisy bearing vibration data. Denoising auto-encoder (DAE) is an improved version of AE, which can enhance the generalization ability and robustness of the AE model and prevent over-fitting problems by adding noise into the input data. Hence, considering the merits of both DAE and VAE, in this section, the concept of variational denoising auto-encoder (VDAE) is presented by integrating a denoising module into the original VAE architecture, which can extract the robust features from the noisy vibration signal. Figure 2 shows the architecture of the VDAE model. Specifically, in the proposed VDAE, based on a binomial distribution (see Equation (6)), noise is firstly added into the original input data $x=\{x_{1},x_{2},\cdots ,x_{n}\}$ to form the corrupted input data $\hat{x}=\{{\hat{x}}_{1},{\hat{x}}_{2},\cdots ,{\hat{x}}_{n}\}$. Subsequently, the corrupted input data layer is connected with the hidden layer of VAE to automatically learn the latent features z=μ+ε⋅σ, where ε represents an auxiliary variable with normal distribution *N*(0, 1),μ and σ denote the features learned from the approximate posterior distribution $q_{\varphi }(z\left|x\right.)$. Finally, the learned latent features, *z,* are connected with the decoder to generate the new data, *y*, which is highly similar to the original data, *x.*

(6) $$\hat{x}\sim q_{D}(\hat{x}\left|x\right.)$$

where $\hat{x}$ denotes the noisy input data and $q_{D}$ represents the binomially distributed random noise.

### 3.2. Stacked Variational Denoising Auto-Encoder

**Figure 3:**
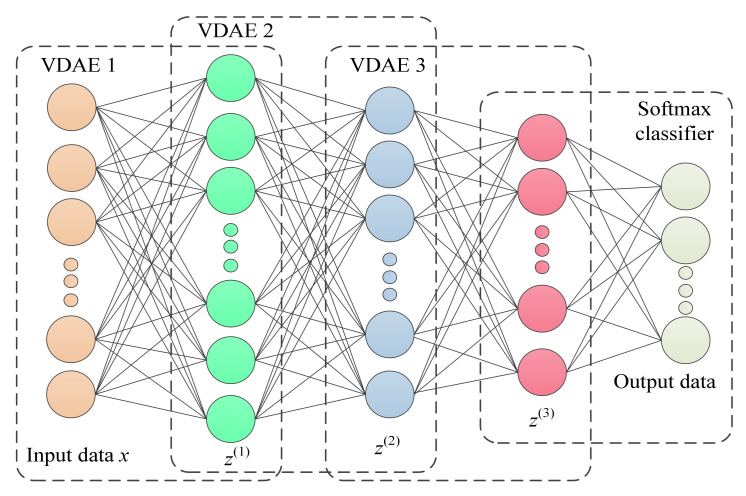
Architecture of the SVDAE model.

Stacked auto-encoder (SAE) is a deep network structure composed of multiple standard AE stacking, which can learn fault characteristics in depth and reduce the dimensionality of input data. Hence, inspired by SAE and VDAE, in this section, a stacked variational denoising auto-encoder (SVDAE) is constructed to extract the latent variables layer by layer. Compared with single VDAE models, SDVAE can extract more robust and more representative latent features due to its deep network structure. Specifically, the training process of the SVDAE model is divided into two stages (i.e., unsupervised pre-training and supervised fine-tuning). Figure 3 shows the architecture of the SVDAE model. In the unsupervised pre-training process of the SVDAE model, the output (i.e., latent variables) of the previous VDAE will be regarded as the input of the next SVDAE to conduct the training process of the SVDAE model. When all VDAE models are trained well, the pre-training process of the SVDAE model is completed, and the representative latent features can be obtained, which is regarded as the input of the classifier. The pre-training process of SVDAE can obtain the local optimal solution of model parameters and reduce the effect of gradient dispersion. In the supervised fine-tuning process of the SVDAE model, the back propagation algorithm is adopted to adjust the network parameters of SVDAE, which is aimed at updating the model parameters. At the end of the SVDAE model, the softmax classifier is adopted to output the identification result in terms of the probability $p{\left(\theta \right)}_{j}$ of Equation (7).

(7) $$p{\left(\theta \right)}_{j}=\frac{e^{\left(\theta^{\left(j\right)}x\right)}}{\sum_{k=1}^{K}e^{\left(\theta^{\left(k\right)}x\right)}},\;\begin{array}{c} j=1,2,\cdots ,n \end{array}$$

where $p{\left(\theta \right)}_{j}$ denotes the probability of the *j*-th class fault, *x* represents the input sample, *K* is the number of fault categories, θ denotes the parameters learned from SVDAE.

### 3.3. Optimized Stacked Variational Denoising Auto-Encoder

**Figure 4:**
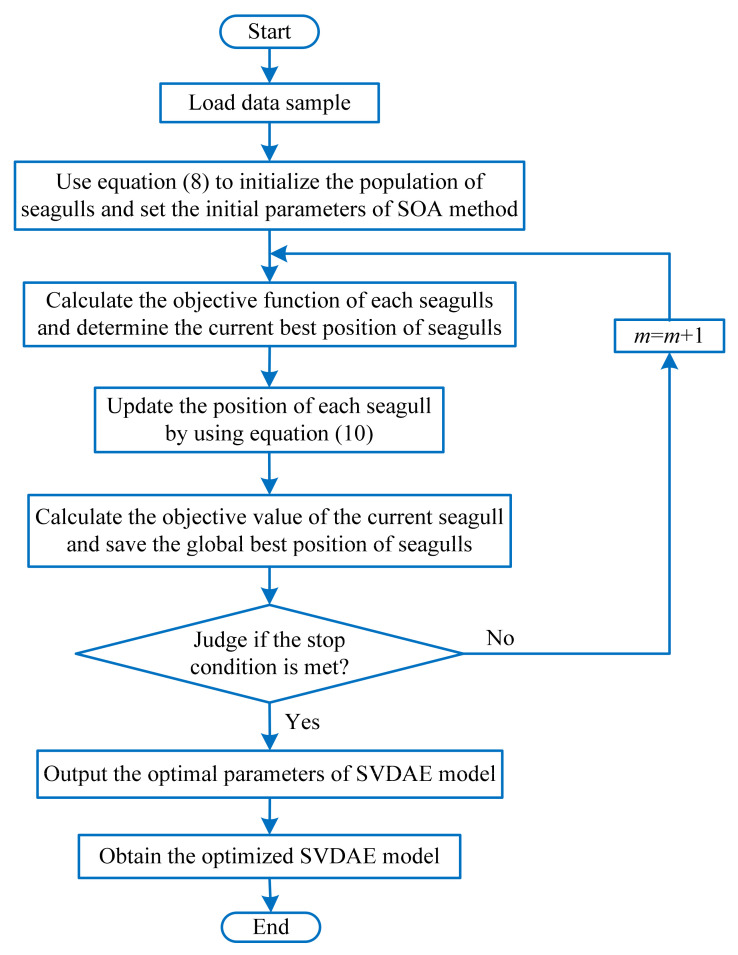
The flowchart of using SOA to optimize the parameters of SVDAE.

When the SVDAE model is used to analyze the collected bearing vibration data, its performance may be affected by the hyper-parameter settings of SVDAE. That is, improper network model parameters will result in low recognition accuracy. If artificial adjustment methods are adopted to select the parameters of SVDAE, it will be tedious and complex, while high accuracy cannot be fully guaranteed. This indicates that an intelligent algorithm is urgently needed to determine the parameters of SVDAE. The seagull optimization algorithm (SOA) is a novel meta-heuristic optimizer proposed by Dhiman and Kumar, in 2019 [17]. Compared with several well-known meta-heuristics (e.g., spotted hyena optimizer (SHO), grey wolf optimizer (GWO), particle swarm optimization (PSO), sine cosine algorithm (SCA) and genetic algorithm (GA)), SOA has the shorter running time and can converge more rapidly towards the expected position because of its adaptive mechanism. Therefore, in this paper, the SOA method is introduced to automatically determine several important parameters (i.e., number of iterations *N*, learning rate *a*, number of nodes in the first hidden layer *l*_1_, number of nodes in the second hidden layer *l*_2_ and number of nodes in the third hidden layer *l*_3_) of the SVDAE model, which can improve its identification accuracy and avoid the problem of artificial empirical selection of the SVDAE model’s parameters. Figure 4 shows the flowchart of using the SOA to optimize the parameters of the SVDAE model. The specific optimization procedures are expressed as follows:

(1) Load sample data, use Equation (8) to initialize the population of seagulls and set the initial parameters of the SOA. Specifically, set the population size of seagulls *N* = 100, maximum number of iterations $Max_{iteration}$ = 200, the frequency coefficient $f_{c}$ = 2, the current iteration number x=1 and the constants μ=υ=1 Considering there are five variables in the SVDAE model that need to be optimized, the position of each seagull is expressed by a vector ${\vec{P}}_{s}=[N,\alpha ,l_{1},l_{2},l_{3}]$ where α represents the learning rate of each VDAE, *N* denotes the epochs number of the SVDAE model, *l*_1_, *l*_2_ and *l*_3_ are the number of nodes of the hidden layers of VDAE1, VDAE2 and VDAE3, respectively.
  (8) $${\vec{P}}_{s}={\vec{P}}_{\min}+\mathrm{rand}({\vec{P}}_{\max}-{\vec{P}}_{\min}).$$
  where ${\vec{P}}_{\min}$ = [10, 0.001, 400, 200, 50] and ${\vec{P}}_{\max}$ = [200, 1, 800, 400, 100] represent the upper and lower bounds of the vector ${\vec{P}}_{s}$ respectively.
(2) Calculate the objective function of each seagull and determine the current best position of the seagulls. In this step, the identification accuracy f(i) is selected as the objective function, as shown in Equation (9).
  (9) $$\left\{\begin{array}{l} \max\;f(N,\alpha ,l_{1},l_{2},l_{3})=1-\frac{x_{i}}{x_{c}+x_{i}}\\ \begin{array}{ll} s.t & \begin{array}{l} N\in [10,200],\alpha \in [0.001,1],\\ l_{1}\in [400,800],l_{2}\in [200,400],l_{3}\in [50,100]\; \end{array} \end{array} \end{array}\right.$$
  where $x_{i}$ denotes the number of misidentified samples, and $x_{c}$ is the number of correctly identified samples.
(3) According to Equation (10), calculate the distance ${\vec{D}}_{s}$ between the current seagull and the best seagull, and update the position of each seagull.
  (10) $$\left\{\begin{array}{l} {\vec{P}}_{s}(x)=({\vec{D}}_{s}\times x^{\prime }\times y^{\prime }\times z^{\prime })+{\vec{P}}_{bs}(x)\\ {\vec{D}}_{s}=\left|{\vec{C}}_{s}+{\vec{M}}_{s}\right|\\ {\vec{C}}_{s}=A\times {\vec{P}}_{s}(x)\\ {\vec{M}}_{s}=B\times ({\vec{P}}_{bs}(x)-{\vec{P}}_{s}(x)) \end{array}\right.$$
  where ${\vec{P}}_{s}(x)$ denotes the current position in the updating of seagulls, ${\vec{P}}_{bs}(x)$ denotes the current best position in the updating of seagulls and *x* denotes the current iteration number. ${\vec{D}}_{s}$ represents the distance between the current seagull and the best seagull, ${\vec{C}}_{s}$ represents the current seagull position that will not collide with other seagulls, *A* represents the movement behavior of seagulls in a given search space and meets $A=f_{c}-(x\times (f_{c}/Max_{iteration}))$, $f_{c}$ is a frequency coefficient that can control the variation range of the variable *A,* which is linearly decreased from $f_{c}$ to 0, and $x=0,1,2,\cdots ,Max_{iteration}$. ${\vec{M}}_{s}$ denotes the position of each seagull moving towards the best seagull, *B* is a random number and satisfies $B=2\times A^{2}\times rd$, rd is a random number between 0 and 1. $x^{\prime }=r\times \cos(k)$, $y^{\prime }=r\times \sin(k)$ and $z^{\prime }=r\times k$ are all plane coordinates, while $r=u\times e^{kv}$ represents the radius of each turn of the spiral, u and v are constants that controls the shape of the spiral and *k* denotes a random number between 0 and 2π.
(4) Calculate the objective value of the current seagull and save the global best position of the seagulls. If ${\vec{P}}_{s}(x)$ is better than ${\vec{P}}_{bs}(x)$, ${\vec{P}}_{s}(x)$ is considered as the global best position of the seagulls. Otherwise, ${\vec{P}}_{bs}(x)$ is considered the individual best position of seagulls from which to continue to update.
(5) Judge if the stop condition is met. Specifically, judge whether the number of iterations is reached or the expected identification accuracy is achieved. If it reaches maximum number of iterations, output the best position ${\vec{P}}_{bs}(x)$ of the seagulls (i.e., the optimal combination parameters of the SVDAE model). Otherwise, set *m* = *m* + 1, repeat the steps (2)~(5) until the stop condition is satisfied.
(6) Use the optimized SVDAE model to automatically learn the discriminative fault feature information from the collected bearing vibration signal.

**Figure 5:**
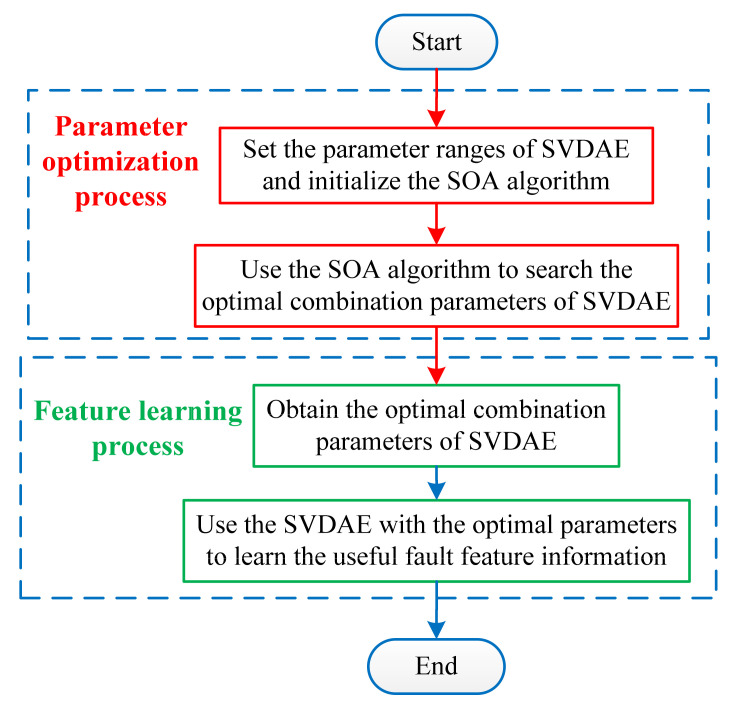
The block diagram of the proposed OSVDAE.

To make it easier for the reader to understand, we plotted the block diagram of OSVDAE, as shown in Figure 5. Specifically, the proposed OSVDAE includes two stages (i.e., parameter optimization process and feature learning process), wherein the first stage is regarded as the parameter optimization process of using SOA to determine the optimal combination parameters of SVDAE, and the second stage is regarded as the bearing fault feature learning process of using the OSVDAE model.

### 3.4. Proposed OSVDAE-Based Fault Diagnosis Method

**Figure 6:**
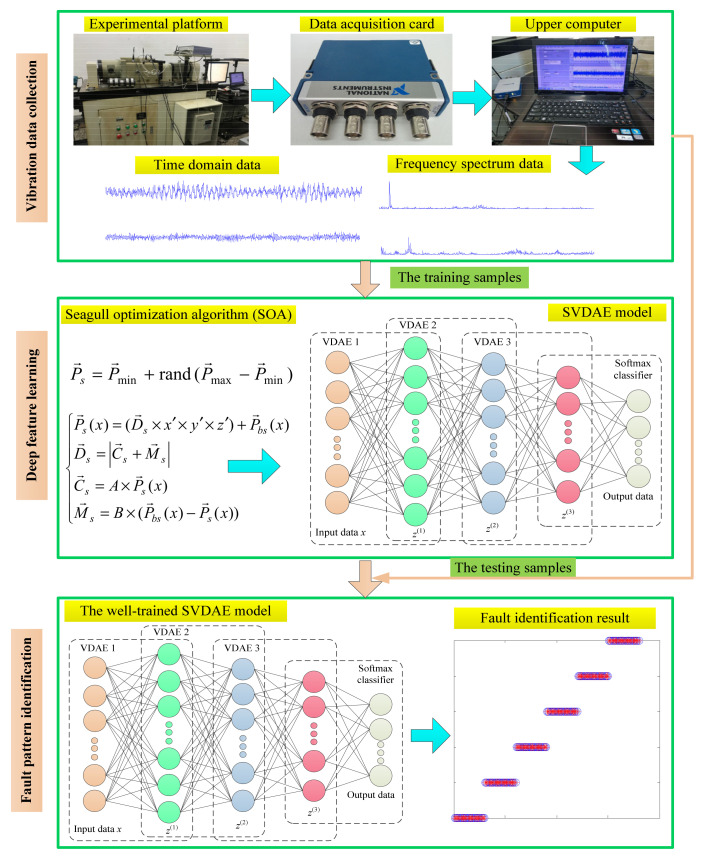
The flowchart of the proposed fault diagnosis method.

To extract more robust fault features from bearing vibration data and improve fault identification accuracy, this paper proposes a bearing fault diagnosis scheme based on optimized stacked variational denoising autoencoder (OSVDAE) with the seagull optimization algorithm (SOA), which can avoid the problem of complexity and the triviality of manually adjusting the parameters of the SVDAE model. Figure 6 shows the flowchart of the proposed method for the fault diagnosis of rolling bearings, which is primarily composed of three steps (i.e., vibration data collection, deep feature learning and fault pattern identification) and its general procedure is expressed as follows:

Step 1: Vibration Data Collection. Bearings’ vibration data are collected by using a data acquisition card and an accelerometer mounted on an experimental platform. Additionally, a fast Fourier transform (FFT) of the collected bearing vibration data is conducted to obtain the frequency spectrum data, then their frequency spectra are randomly selected as training samples or testing samples.

Step 2: Deep Feature Learning. The training samples are input into the constructed OSVDAE model to train the stacked encoding network layer-by-layer, which is aimed at obtaining the representative latent fault features. Meanwhile, the SOA method is employed to automatically determine the important parameters of the OSVDAE model.

Step 3: Fault Pattern Identification. The testing samples are fed into the well-trained OSVDAE model to automatically identify bearing faults and provide the final intelligent bearing fault diagnosis.

## 4. Experimental Verification and Discussion

### 4.1. Case 1: Bearing Experimental Data from CWRU

#### 4.1.1. Data Description and Settings

**Figure 7:**
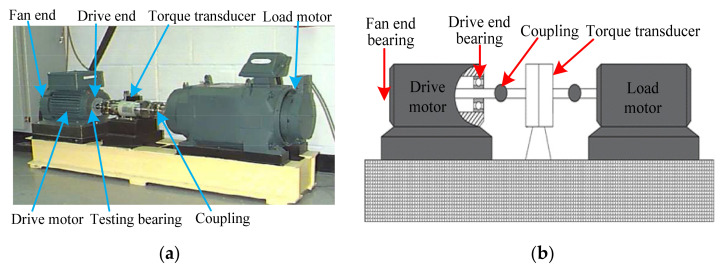
(**a**) The experimental equipment and (**b**) its corresponding structure diagram.

**Figure 8:**
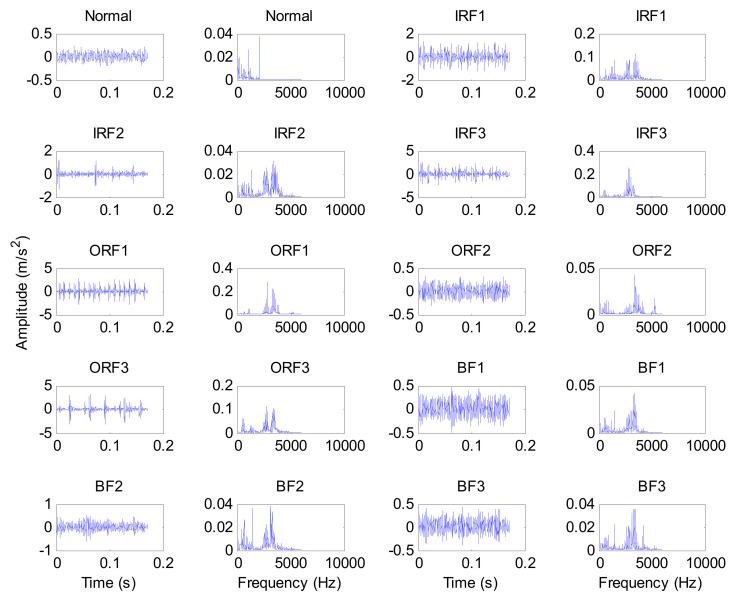
Time-domain waveform and frequency spectrum of one data sample from each kind of bearing condition.

Experimental data are provided by the Bearing Data Center of Case Western Reserve University (CWRU). The experimental apparatus mainly consists of a motor, testing bearing, a torque transducer, a dynamometer and control electronics. Figure 7 shows photos of the experimental apparatus and a corresponding structure diagram thereof. Table 2 lists the specific parameters of the experimental bearing. In this experiment, single point bearing faults (i.e., inner race fault (IRF), outer race fault (ORF), ball fault (BF)) are introduced to the testing bearings using electro-discharge machining, where each kind of fault has three fault diameters of size 7 mils, 14 mils and 21 mils (1 mil = 0.001 inches). Additionally, the damage point of the bearing’s outer race fault is oriented to six o’clock. In the data collection process, 10 kinds of bearing vibration data (see Table 3), under different motor speeds, are obtained by using the motor drive end sensor with a sampling frequency of 12 kHz. Concretely, in this paper, normal condition data under 1730 rpm, IRF data of three fault parameters under 1750 rpm, ORF data of three fault parameters under 1772 rpm and BF data of three fault parameters under 1797 r/min are obtained. That is, 10 kinds of bearing working conditions are selected in this paper. Sixty data samples of each bearing’s working conditions are intercepted by using a sliding window with the length of 2048 points. The length of each sample is 2048 data points. Table 2 lists the detailed bearing experimental data adopted in this paper, where 30 samples of each bearing’s working conditions are randomly chosen as the training samples and the remaining 30 samples are considered testing samples. That is, the training-to-testing samples ratio is 1:1. Figure 8 plots the time domain waveform and frequency spectrum of one data sample from each kind of bearing condition. As seen in Figure 8, although there are some differences in the shape of the time domain waveform and spectrum, it is very difficult to accurately judge bearing fault conditions because of the interference from signal noise.

#### 4.1.2. Results and Analysis of Fault Diagnosis

**Figure 9:**
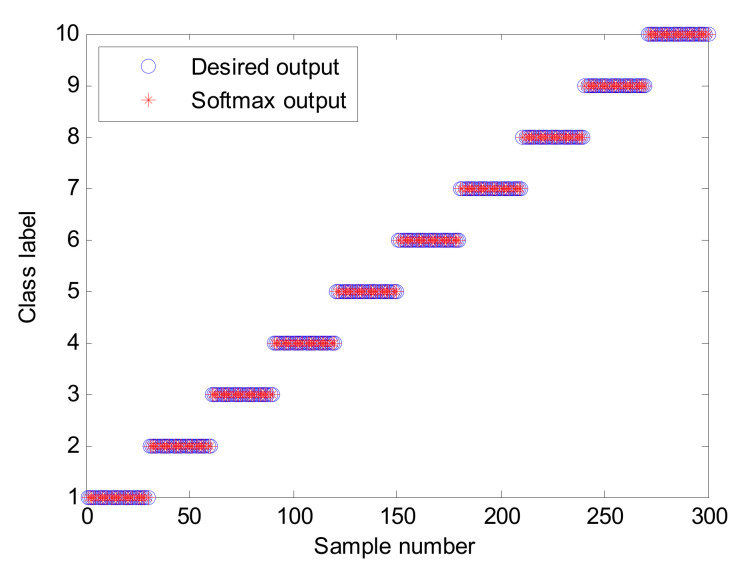
Identification results of the first trial of the proposed method in case 1.

**Figure 10:**
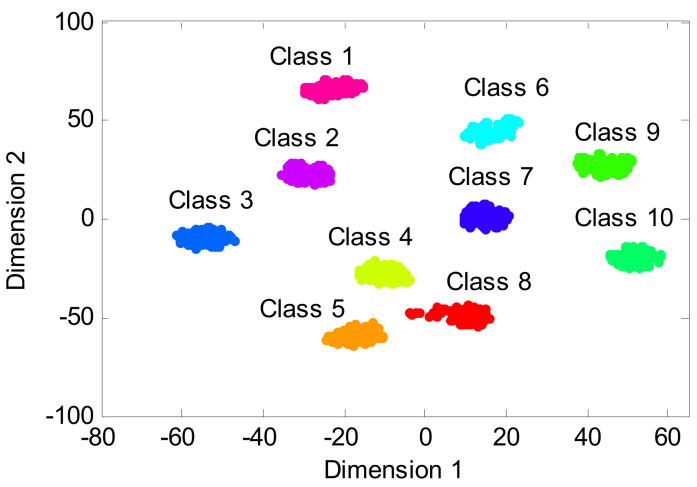
The results of the two-dimension visualization of the output features of the proposed method using t-SNE.

The proposed method is used to process the above collected bearing experimental data, where the key parameters of the SVDAE model are automatically determined by the SOA method. Table 4 lists the optimal combination parameters of the SVDAE model obtained by the SOA method. According to the flowchart of the proposed method, the frequency spectra data matrix of size 300 × 2048 is regarded as the training sample set to train an SVDAE model with optimized parameters, while the remainder frequency spectra data matrix of size 300 × 2048 is considered as the testing sample set with which to test the recognition performance of the well-trained SVDAE model and obtain bearing fault diagnoses. Figure 9 shows the identification accuracy of the proposed method in the first trial. It can be observed from Figure 9 that the proposed method can obtain 100% identification (300/300), which means that the proposed method can effectively identify different bearing fault conditions under variable speeds. In addition, to intuitively observe the output features of the proposed method and prove the superiority of its feature learning performance, a feature visualization method, t-distributed stochastic neighbor embedding (t-SNE), is adopted to map the high-dimensional features learned by the third hidden layer of the proposed method into two-dimension space. Figure 10 shows the results of the two-dimension visualization of the output features of the proposed method using t-SNE. It can be seen from Figure 10 that the output features learned by the proposed method have an obvious degree of discrimination, which is the reasons for selecting the proposed method for bearing fault diagnosis. It is also verifies that the proposed method has good feature learning ability.

**Figure 11:**
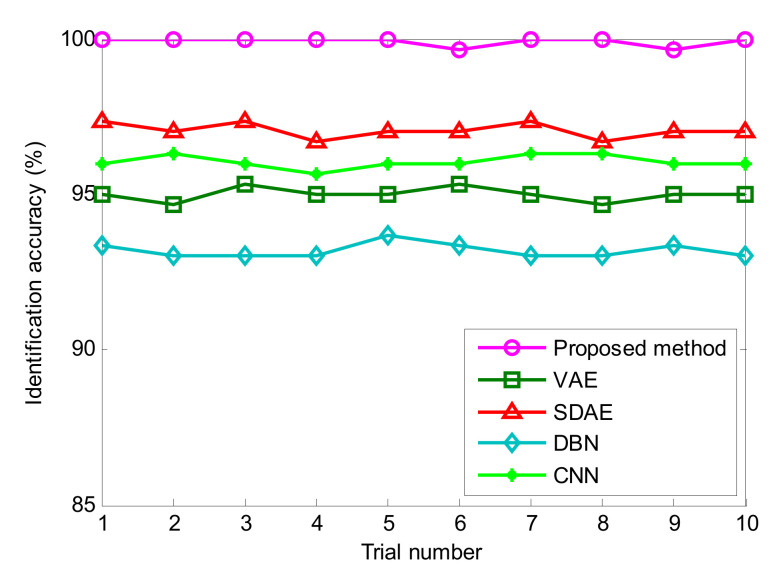
Identification accuracy obtained by different methods for 10 trials in case 1.

To show the advancement of the proposed method and enhance the reliability of the diagnosis results, the same experimental data from above are analyzed by the traditional VAE and three representative deep learning models (i.e., SDAE, DBN and CNN). Due to the randomness of sample selection, each algorithm is executed 10 times to observe their identification results. According to previous studies, their model parameters are specifically summarized as follows: (1) In the traditional deep VAE [30], the network structure is set as 2048–512–64–32–10, which includes one input layer, three hidden layers and one output layer. (2) In the SDAE model [31], the network structure is set as 2048–200–100–80–10, which includes one input layer, three hidden layers and one output layer. (3) In the DBN model [32], the network structure is set as 2048–200–100–80–10, which includes one input layer, three hidden layers and one output layer. (4) In the CNN model [33], the network structure includes one input layer, two convolutional layers, two pooling layers and one fully connected layer, where the size of the input layer is 32 × 32, the convolution kernel sizes of two convolutional layers are, respectively, 4 and 3, the size of both pooling layers is 2. (5) For a fair comparison, the number of iterations, or the learning rate, of the SDAE, DBN and CNN models is selected as the same as that of the proposed method. Figure 11 shows the identification results of different deep learning methods in 10 trials. Additionally, Table 5 provides the detailed diagnosis results of different models, including maximum, minimum, mean and standard deviation of identification accuracy. As seen from Figure 11 and Table 5, the average recognition accuracy of the proposed method (OSVDAE) is 99.93%, which is obviously higher than that of the other four methods (VAE, SDAE, DBN and CNN), which are 95.00%, 97.03%, 93.17% and 96.06%, respectively. This verifies that the proposed method is effective in discriminating different bearing faults at unsteady speeds. In other words, the proposed method reliably diagnoses rolling bearing faults.

**Figure 12:**
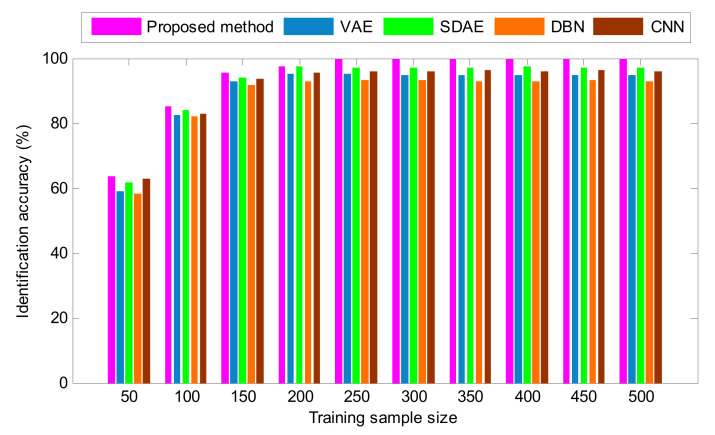
The identification results of various methods for different training sample sizes in case 1.

To study the impact of training sample size on the diagnosis ability of different deep learning methods, the total training sample size is respectively set to 50, 100, 150, 200, 250, 300, 350, 400, 450 and 500, where the number of training samples of each bearing condition is 10% of the total size of the training sample data. The identification results of different methods are plotted in Figure 12. As shown in Figure 12, the recognition accuracy of different methods increases with more training samples, which means that sufficient training samples are necessary for using these methods to achieve a high identification accuracy. Generally, the more training samples, the better trained is the model and the higher its diagnostic accuracy. However, a large training sample size will result in a large amount of computation, so this work selects the same training and testing sample size to strike a balance between identification accuracy and computational efficiency. That is, the ratio of training to testing samples is 1:1. Additionally, it can also be seen from Figure 12 that the proposed method can obtain a higher identification accuracy than the other methods, even if the training sample size is different. That is, the validity of the proposed method is further verified.

**Figure 13:**
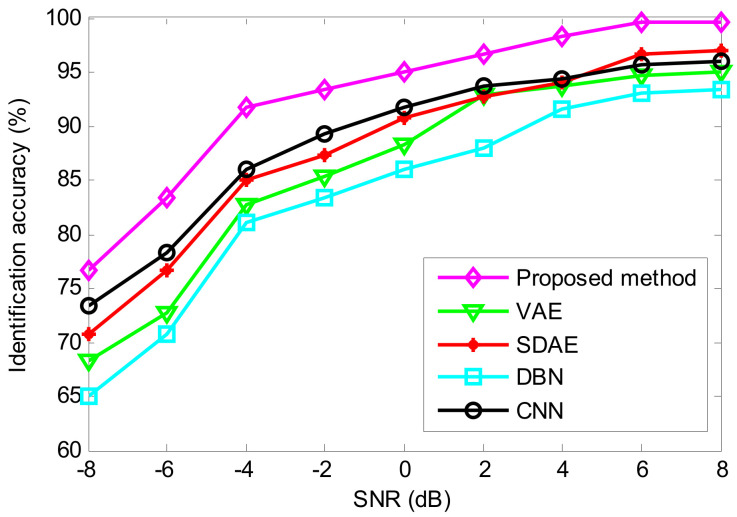
Identification accuracy of the proposed method under different SNR conditions in case 1.

To further consolidate the diagnosis results, we analyze the impact of noise on the recognition performance of the proposed method. Concretely, Gaussian white noise of different signal-to-noise ratios (SNR) is added into the original vibration data to investigate and compare the results of the various deep learning methods. Figure 13 shows the identification results of various deep learning methods at different noise levels (i.e., SNR = −8 dB, −6 dB, −4 dB, −2 dB, 0 dB, 2 dB, 4 dB, 6 dB, 8 dB). As seen from Figure 13, as the intensity of additional noise increases, the identification accuracy of various deep learning methods follows a trend of decline from SNR = 6 dB to −8 dB. When the added noise level is greater than 6 dB, the various methods gradually achieve a steady accuracy of 90% above. Additionally, among these comparison methods, the proposed method achieves the best identification accuracy. This indicates that the proposed method has the most robustness against noise and performs better than the other four methods (VAE, SDAE, DBN and CNN) in bearing fault identification.

To more deeply investigate the diagnostic results of the proposed method, the five-fold cross-validation of different deep learning methods is conducted. Concretely, the collected dataset (600 samples) is equally divided into five parts (i.e., each part has 120 samples), where four parts (i.e., 480 samples) are alternately selected as the training samples and the remaining part (i.e., 120 samples) is regarded as the testing samples. That is, five trials of different methods are performed. The average identification accuracies of the five trials are regarded as the final diagnoses. Table 6 lists the diagnosis results of different methods with five-fold cross validation. As seen from Table 6, the average identification accuracies (i.e., the proposed method, VAE, SDAE, DBN and CNN) of five methods are 99.83%, 95.49%, 97.16%, 92.99% and 96.33%, respectively. Obviously, the proposed method can obtain a greater recognition accuracy than the other four methods (VAE, SDAE, DBN and CNN), which once again indicates that the proposed method has a better bearing fault recognition performance.

### 4.2. Case 2: Bearing Vibration Data from Laboratory

#### 4.2.1. Data Description and Settings

**Figure 14:**
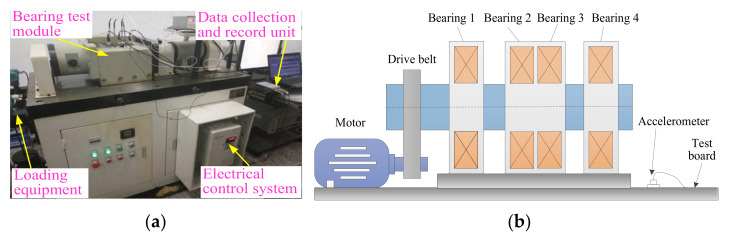
(**a**) Rolling bearing test rig and (**b**) its structure schematic diagram.

**Figure 15:**
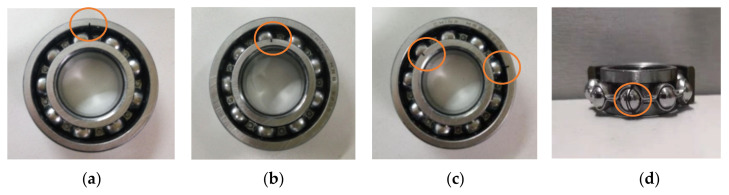
Photo of the faulty bearing: (**a**) ORF, (**b**) IRF, (**c**) OIRCF and (**d**) BF.

**Figure 16:**
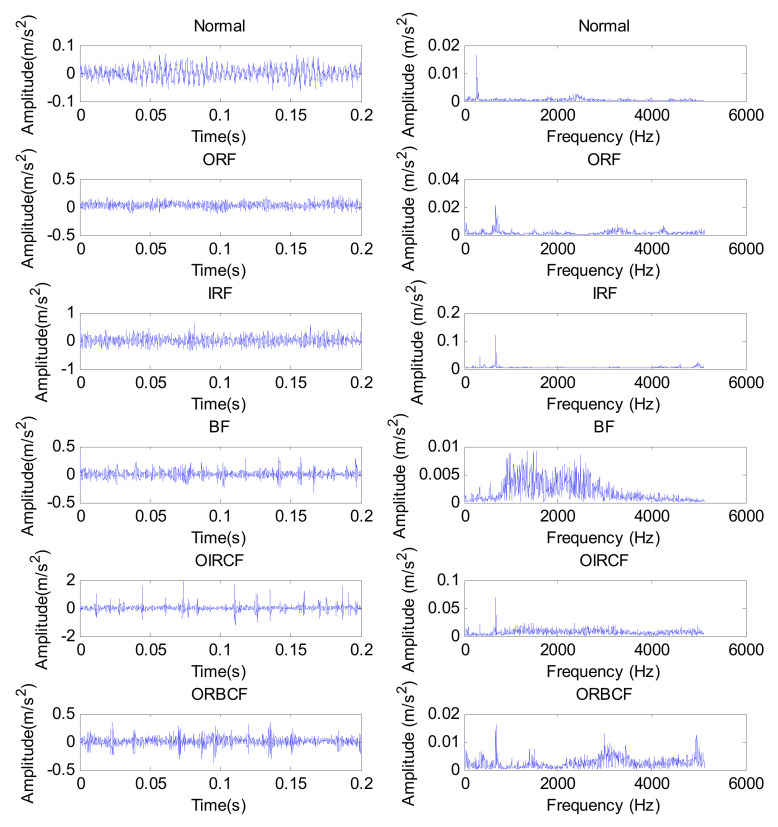
Time domain waveform and frequency spectra from one data sample each of the different bearing conditions, in case 2.

In this case, an experimental bearing dataset from the laboratory is used to evaluate the performance of the proposed method. The experimental equipment mainly consists of a motor, drive belt, loading equipment, a bearing test module, an electrical control system and a computer monitoring system, which can be seen in Figure 14. The bearing test module includes four bearings of model HRB6205, and Table 7 lists the detailed specifications of the testing bearings. In this experiment, five types of faults (i.e., inner race fault (IRF), outer race fault (ORF) and ball fault (BF), outer and inner race compound fault (OIRCF), outer race and ball compound fault (ORBCF)) are introduced to normal bearings using electro-discharge machining. Figure 15 shows a global view of a faulty bearing. With the motor running at 1050 rpm, a PCB accelerometer with a sensitivity of 100 mV/g is mounted on near bearing case to collect bearing vibration data of different conditions. The adopted data collection and recording unit is a NI9234 acquisition card with four input channels. The sampling frequency of the signal is set to be 10,240 Hz. The collected bearing vibration data are intercepted into 480 samples (the length of each sample is 2048 points) using a sliding window, from which 40 samples of each bearing condition are randomly selected as the training set and the remaining samples are regarded as the testing set. That is, the sizes of the training and testing sets are both 240 (i.e., the ratio is 1:1). Table 8 displays the specific description of the adopted bearing experimental data. Figure 16 plots time domain waveform and frequency spectra of one data sample from each bearing condition. As seen from Figure 16, although there are some differences in the waveforms and spectra of the vibration data between bearing conditions, it is very difficult and impractical to manually identify bearing fault conditions by observing their differences, especially for a significant amount of real bearing vibration data containing environmental noise. Hence, a new method for reliably diagnosing fault in rolling bearings is urgently needed.

#### 4.2.2. Results and Analysis of Fault Diagnosis

**Figure 17:**
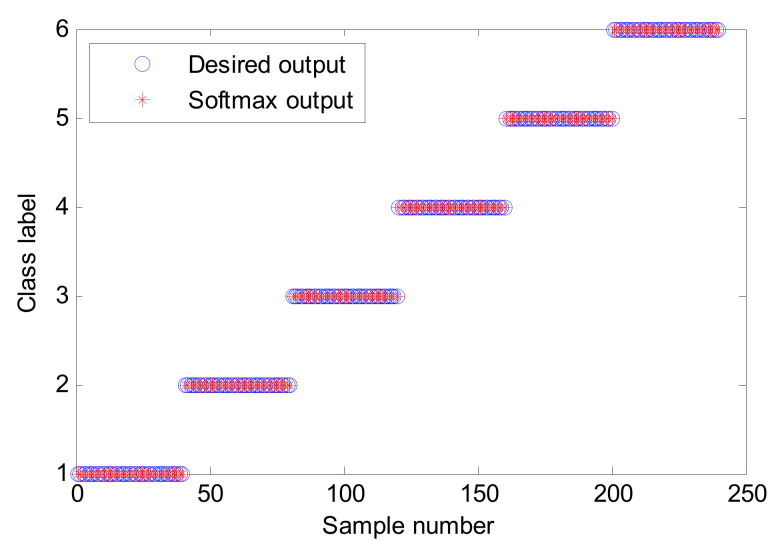
Identification results of the first trial of the proposed method in case 2.

The proposed method was adopted to identify the above bearing fault conditions. According to the process of the proposed method, the SOA method is firstly used to automatically determine the important parameters of the SVDAE model. The optimized results are shown in Table 9. Then, the optimized SVDAE model containing the optimal parameters is trained by the training sample matrix of size 240 × 2048 in the frequency domain, while the testing sample matrix of the same size is utilized to test the well-trained SVDAE model and output the final fault diagnoses. Figure 17 shows the identification result of the proposed method in the first experiment. As is apparent, the proposed method achieves a 100% testing accuracy (240/240), which indicates that the proposed method is effective in identifying bearing fault types.

**Figure 18:**
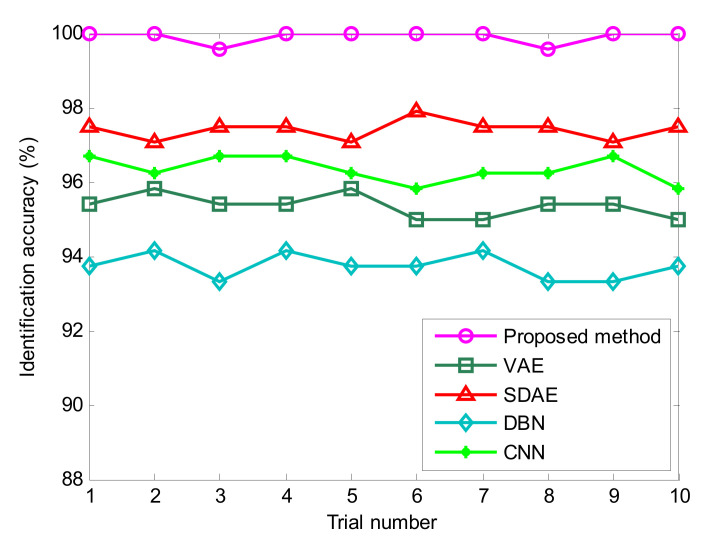
Identification accuracy obtained by different methods for 10 trials in case 2.

To verify the advantages of the proposed method, the same bearing data are also processed using four typical deep learning methods (i.e., VAE, SDAE, DBN and CNN). Moreover, to improve the reliability of the diagnostic results, 10 trials of each method are carried out for a fair comparison. Note that, in all compared methods, the number of iterations and the learning rate are the same as that of the proposed method. The VAE, SDAE and DBN models all have five layers (i.e., one input layer, three hidden layers and one output layer), their network structures are 2048–512–64–32–6, 2048–200–100–80–6, and 2048–200–100–80–6, respectively. The network structure of CNN is set to the same as in case 1. Figure 18 shows the diagnosis results of different methods in 10 trials. Additionally, the maximum, minimum, mean and standard deviation of identification accuracy of different methods are shown in Table 10. It can be observed from Figure 18 and Table 10 that average accuracy (i.e., 99.91%) of the proposed method is the greatest among all the compared deep learning models, while the standard deviation (i.e., 0.1771) of the proposed method is less than that of the comparison methods. Therefore, by this comparison, it can be easily concluded that the proposed method achieves better diagnostic performance and has good stability in identifying bearing fault conditions.

**Figure 19:**
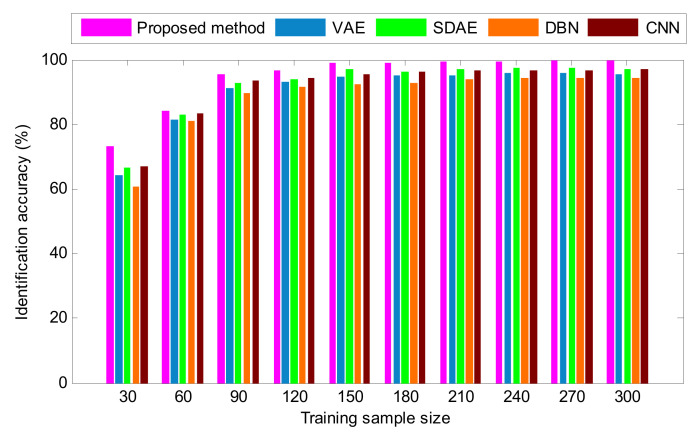
The identification results of various methods for different training sample size in case 2.

To consolidate the diagnostic results of the proposed method, the identification results of various methods under different training samples are calculated, as shown in Figure 19. As seen from Figure 19, as the number of training samples increases, the fault recognition performances of the various deep learning methods gradually follow. Compared with other methods (i.e., VAE, SDAE, DBN and CNN), the proposed method achieves the highest accuracy under almost all sample training scenarios. Theoretically, more training samples represents a better-trained model, but it also increases the training time. Therefore, this worm set the training data and testing data to be of the same size for the purposes of balancing identification accuracy and algorithm efficiency. Additionally, according to Figure 19, a conclusion can be drawn that the proposed method still performs better under varying samples except for the case of training sample size = 30 and 60. That is, the effectiveness and superiority of the proposed method in bearing fault identification is verified due to its denoising mechanism and the parameter optimization technique introduced into the stacked VAE.

**Figure 20:**
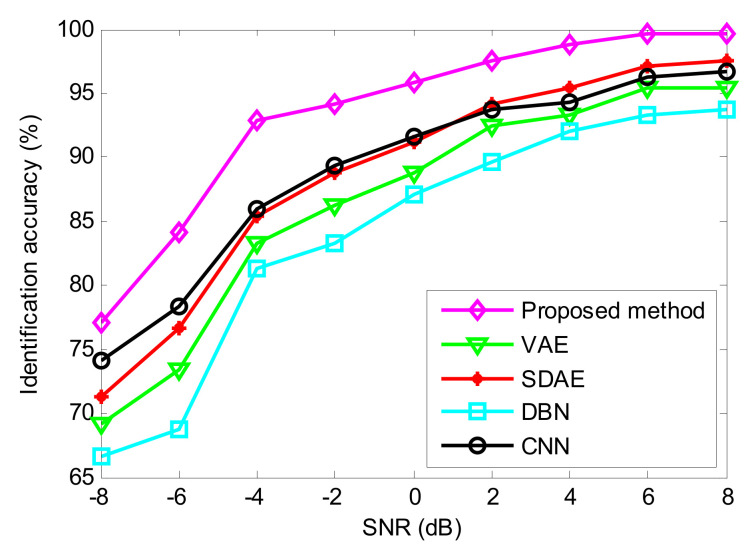
Identification accuracy of various methods at different SNR conditions in case 2.

To investigate the robustness of the proposed method for bearing vibration signals in a noisy environment, we adopt different methods to process the above bearing condition data containing different noise levels (i.e., SNR = −8 dB, −6 dB, −4 dB, −2 dB, 0 dB, 2 dB, 4 dB, 6 dB, 8 dB). Figure 20 shows the identification results of various method at different noise levels. As seen from Figure 20, the identification accuracy of the proposed method increases with increasing signal-to-noise ratio. Additionally, the proposed method can obtain a higher identification accuracy than other state-of-the-art methods (i.e., VAE, SDAE, DBN and CNN), even if the SNR is low. That is, the diagnosis performance of the proposed method is superior, overall compared with the diagnostic performance of the other methods.

To more deeply show the diagnostic results of the proposed method, we conducted five-fold cross-validation on the proposed method and four comparison methods (VAE, SDAE, DBN and CNN). Similarly, the collected dataset are equally divided into five parts, whereby four parts (i.e., 384 samples) are alternately selected as the training set and the remaining part (i.e., 96 samples) is regarded as the testing set. That is, each method conducts five trials. The average accuracy of five trials of each method is regarded as their final diagnosis results (see Table 11 in detail). It can be clearly seen from Table 11 that the proposed method achieves an average identification accuracy of 99.79%, which is obviously higher than those of other four methods (VAE, SDAE, DBN and CNN), which are 95.41%, 97.49%, 93.33% and 96.45%, respectively. This, once again, shows that the proposed method is effective in identifying different bearing fault types and sizes. In other words, the proposed method has a better bearing fault diagnosis ability compared with VAE, SDAE, DBN and CNN.

### 4.3. Research Limitations and Future Work

Based on the comparisons and analysis of fault diagnosis results obtained by different methods under two sets of experimental bearing data, we can draw a definite conclusion that the proposed method has an accurate classification result and has better identification accuracy than several typical deep learning methods mentioned in this paper; this is probably due to the stacked denoising techniques and the parameter optimization process introduced into the original VAE model, which are also the advantages of the proposed method. The proposed method can provide a reference for algorithm the improvement and future study of the VAE model in bearing fault diagnosis. Despite this, this paper nonetheless leaves some work to be done on the proposed method in the future. Some limitations and specific future works of the proposed method are envisaged as follows:

(1) In this paper, through the fusion of stacked denoising techniques, VAE and an optimization algorithm, the proposed method could improve the anti-noise performance of the VAE model and adaptively select its parameters. However, the over-fitting problem of the proposed method was not considered completely. That is, the overfitting problem of the network model is one limitation of this study. Therefore, to solve this problem, in a future work, considering the advantages of the existing available advanced AE (e.g., deep sparse auto-encoder [34], deep contractive auto-encoder [35], deep convolutional auto-encoder [36], wavelet auto-encoder (WAE) [37], and discriminative manifold regularized auto-encoder (DMRAE) [38]), we intend to add a sparsity constraint to the neurons in the hidden layer of the SVDAE and improve the loss function of the SVDAE by adding the Jacobian norm to develop an ensemble stacked variational auto-encoder (ESVAE) that can both prevent over-fitting and improve feature-learning ability.
(2) It is difficult to obtain sufficient bearing fault data in actual production, which easily leads to imbalanced samples from training. This indicates that bearing fault diagnosis, under data imbalance, is a difficult problem. That is, another limitation of this study is the uncertainty of implementing unbalanced bearing fault diagnosis. Therefore, to solve the unbalanced fault diagnosis problem, in a future work, we intend to integrate the synthetic minority over-sampling technique or bagging method into SVDAE to improve the generalization performance of the network model. Additionally, some loss items (e.g., the focal loss, piecewise loss) can also be added into the SVDAE model to solve the unbalanced fault diagnosis problem of rolling bearings, which is our future research direction.
(3) When multi-sensor experimental data are collected, including vibration and acoustic signals, the utilization of multi-sensor information is very critical for the reliable fault diagnosis of bearings. This paper mainly solves the bearing fault diagnosis problem of single-sensor data, whereas the effectiveness of the proposed method is unknown for multi-sensor data processing. That is, the indeterminacy of the proposed method in multi-sensor fault diagnosis is regarded as one limitation of this study. Therefore, in our future work, we will refer to the coupling notion of a deep coupling auto-encoder (DCAE) [39], by which the proposed method can be improved to handle multi-sensor data, aimed at capturing the joint fault feature information between multi-sensor signals and perform more accurate fault diagnosis.
(4) In the experimental case 2 of this paper, bearing vibration data were actually collected on different dates. As the room temperature was different on different dates, the collected bearing vibration data was collected in different room temperatures. This indicates that the proposed method can identify bearing faults under different room temperatures. In other words, when bearing vibration data of different room temperatures are analyzed, it is generally not required to re-train the proposed model. However, due to the limitations of the experimental conditions, when the experiments of this paper were conducted, the room temperatures were not recorded, which is regarded as one deficiency of this study. Therefore, in our future work, we will adopt a thermodetector to record the room temperature in which bearing data is collected and confirm the identification accuracy of the proposed method as a function of room temperature.
(5) In two experimental cases of this paper, the robustness and parameter selection of the proposed SVDAE were studied intensively, but the internal working mechanism of the SVDAE was not investigated in detail. Therefore, in our future work, we will adopt some representative feature visualization techniques (e.g., t-distributed stochastic neighbor embedding (t-SNE), principal component analysis (PCA), local preserving projection (LPP) and local sensitive discriminant analysis (LSDA)) to clearly describe the clustering of output features of the different layers of SVDAE. Meanwhile, in a future work, the discrimination degree of the output features of the proposed method will be quantitatively evaluated by using sensitive indexes (e.g., intra-class distance, inter-class distance and the ratio between them).

## 5. Conclusions

This paper proposed a novel bearing fault diagnosis scheme based on optimized stacked variational denoising autoencoder (OSVDAE). Two experimental examples were adopted to demonstrate the effectiveness of the proposed method in identifying bearing faults. The experimental comparison results showed that the proposed method could obtain a high identification accuracy of 99% or above, which was greater than those of the traditional VAE and several representative deep learning models (i.e., SDAE, DBN and CNN). Additionally, the proposed method showed certain robustness for bearing fault signals in noisy environments. The main contributions and novelties of this paper are as follows:

(1) A novel deep learning model, named stacked variational denoising auto-encoder (SVDAE), was presented to extract the inherent fault features from the original bearing vibration data and provided better robustness than the original VAE.
(2) A new meta-heuristic intelligent optimizer, termed the seagull optimization algorithm (SOA), was employed to automatically select the hyper-parameters of SVDAE model, which improved the feature learning ability and avoided the dependence upon human experience for parameter selection in deep learning.
(3) An intelligent diagnostic framework based on optimized stacked variational denoising autoencoder (OSVDAE) was proposed for reliably diagnosing bearing faults that does not require artificial, features-based expert knowledge and so is appropriate for ordinary technicians.
(4) The experimental results and comparison analysis of bearing fault diagnosis showed the effectiveness and superiority of the proposed method.

Due to the limitations of the research conditions, this paper mainly focused on solving the problem of bearing fault diagnosis, whereas other key parts (e.g., gear, rotor and blade) of mechanical equipment are also prone to breaking down. Hence, in future work, the problem of reliable fault diagnosis in other key parts (e.g., gear, rotor and blade) will be analyzed using the proposed method.

## Figures and Tables

**Table 1:** Summary of VAE in fault diagnosis.

Authors	Year	Country/Region	Research Problem	Methods	Experiment Validation (Yes/No)	Data Access
Chen et al.	2019	China	fault diagnosis of diesel engine	VSAE	yes	private
Huang et al.	2019	Taiwan	motor fault detection	RNN-based VAE	yes	private
Martin et al.	2019	Chile	bearing fault diagnosis	Deep VAE	yes	public
Zhao et al.	2020	China	bearing fault diagnosis	VAE and CNN	yes	private
Wang et al.	2020	China	fault diagnosis of planetary gearbox	CVAE-GAN	yes	private
Yan et al.	2020	China and Singapore	chiller fault diagnosis	CWGAN-GP-VAE	yes	private
Tang et al.	2021	China	quality-related fault detection	VAE and VIB	yes	private
Costa et al.	2021	Spain	visual diagnosis of atrial fibrillation	RVAE	yes	private
Kim et al.	2021	Republic of Korea	semiconductor fault detection	VAE model	yes	private
Zhang et al.	2021	USA	bearing fault diagnosis	VAE-based deep generative models	yes	public
Chao et al.	2021	Switzerland	fault diagnosis of safety-critical systems	KIL-AdaVAE	yes	public

**Table 2:** Specifications of experimental bearing in case 1.

Bearing Type	Roller Diameter (mm)	Pitch Diameter (mm)	Number of Balls	Contact Angle (^o^)
SKF6205-2RS	7.94	39.04	9	0

**Table 3:** Bearing experimental data description in case 1.

Bearing Condition	Motor Speed (rpm)	Fault Diameter (mils)	Number of Training Samples	Number of Testing Samples	Class Label
Normal	1730	0	30	30	1
Inner race fault 1 (IRF1)	1750	7	30	30	2
Inner race fault 2 (IRF2)	1750	14	30	30	3
Inner race fault 3 (IRF3)	1750	21	30	30	4
Outer race fault 1 (ORF1)	1772	7	30	30	5
Outer race fault 2 (ORF2)	1772	14	30	30	6
Outer race fault 3 (ORF3)	1772	21	30	30	7
Ball fault 1 (BF1)	1797	7	30	30	8
Ball fault 2 (BF2)	1797	14	30	30	9
Ball fault 3 (BF3)	1797	21	30	30	10

**Table 4:** The optimal parameters of SVDAE in case 1.

Model Parameters	Value
The number of iterations N	180
The learning rate *a*	0.009
The number of nodes in the first hidden layer *l*_1_	660
The number of nodes in the second hidden layer *l*_2_	250
The number of nodes in the third hidden layer *l*_3_	80

**Table 5:** Diagnosis results of different deep learning methods in case 1.

Different Methods	Identification Accuracy Obtained Using Different Methods (%)			
	Maximum	Minimum	Mean	Standard Deviation
Proposed method	100	99.67	99.93	0.1391
VAE	95.33	94.67	95.00	0.2200
SDAE	97.33	96.67	97.03	0.2435
DBN	93.67	93.00	93.17	0.2357
CNN	96.33	95.67	96.06	0.2087

**Table 6:** Diagnosis results of different methods with five-fold cross validation in case 1.

Different Methods	Accuracy Obtained by Different Methods for Five Trials (%)					Average Accuracy (%)
	1	2	3	4	5	
Proposed method	100	100	99.17	100	100	99.83
VAE	95.83	95.00	95.83	95.83	95.00	95.49
SDAE	97.50	97.50	96.67	96.67	97.50	97.16
DBN	93.33	92.50	93.33	93.33	92.50	92.99
CNN	96.67	96.67	95.83	96.67	95.83	96.33

**Table 7:** Specifications of testing bearing in case 2.

Bearing Type	Ball Diameter (mm)	Pitch Diameter (mm)	Number of Balls	Contact Angle (°)
HRB6205	7.94	39.04	9	0

**Table 8:** The detailed description of bearing experimental data in case 2.

Bearing Conditions	Fault Diameter (Width × Depth)	Number of Training Samples	Number of Testing Samples	Class Labels
Normal	0 × 0 mm	40	40	1
Outer race fault (ORF)	0.1 × 0.6 mm	40	40	2
Inner race fault (IRF)	0.1 × 0.5 mm	40	40	3
Ball fault (BF)	0.1 × 0.6 mm	40	40	4
Outer and inner race compound fault (OIRCF)	0.1 × 0.5 mm	40	40	5
Outer race and ball compound fault (ORBCF)	0.1 × 0.6 mm	40	40	6

**Table 9:** The optimal parameters of SVDAE in case 2.

Model Parameters	Value
The number of iterations N	150
The learning rate *a*	0.003
The number of nodes in the first hidden layer *l*_1_	580
The number of nodes in the second hidden layer *l*_2_	270
The number of nodes in the third hidden layer *l*_3_	60

**Table 10:** Diagnosis results of different deep learning methods in case 2.

Different Methods	Identification Accuracy Obtained Using Different Methods (%)			
	Maximum	Minimum	Mean	Standard Deviation
Proposed method	100	99.58	99.91	0.1771
VAE	95.83	95.00	95.37	0.3058
SDAE	97.91	97.08	97.41	0.2635
DBN	94.16	93.33	93.74	0.3389
CNN	96.67	95.83	96.33	0.3313

**Table 11:** Diagnosis results of different methods with five-fold cross validation in case 2.

Different Methods	Accuracy Obtained by Different Methods for Five Trials (%)					Average Accuracy (%)
	1	2	3	4	5	
Proposed method	100	100	98.95	100	100	99.79
VAE	95.83	95.83	94.79	94.79	95.83	95.41
SDAE	97.91	96.87	97.91	97.91	96.87	97.49
DBN	93.75	93.75	92.71	92.71	93.75	93.33
CNN	96.87	95.83	96.87	96.87	95.83	96.45

## Data Availability

The data used in this study are all owned by the research group and will not be transmitted.
